# Endurance Training Improves Oxygen Uptake and Endurance Capacity in Patients With Moderate to Severe Valvular Disease

**DOI:** 10.3389/fcvm.2021.627224

**Published:** 2021-02-12

**Authors:** Karin Vonbank, Daniel Haubenberger, Raphael Rosenhek, Matthias Schneider, Stefan Aschauer, Marco Idzko, Harald Gabriel

**Affiliations:** ^1^Institute of Internal Medicine II, >Medical University of Vienna, Vienna, Austria; ^2^Department of Gynecology, Hospital Scheibbs, Scheibbs, Austria

**Keywords:** endurance training, valve disease, peak VO_2_, Watt_max_, endurance capacity

## Abstract

**Aim:** Peak oxygen uptake (peakVO_2_) is one of the strongest predictors of survival in patients with valvular heart disease. The purpose of this study was to determine whether endurance training improves peakVO_2_ and endurance capacity in patients with moderate-severe aortic and mitral valve disease.

**Methods:** 30 patients with moderate-severe valvular heart disease were randomly assigned to 12 weeks of endurance training (TG) (*n* = 16) or standard care (SC) (*n* = 14). PeakVO_2_ and maximum working capacity (Watt_max_) were assessed by cardiopulmonary exercise testing, as well as submaximal endurance test at 80% of peakVO_2_ at baseline and after 12 weeks.

**Results:** There was a significant improvement in peakVO_2_ from 27.2 ± 5.9 ml/kg to 30.4 ± 6.3 ml/kg (*P* < 0.001) in TG compared to the SC (peakVO_2_ from 24.6 ± 4.4 to 24.7 ± 3.8) and in the Watt_max_ from 151.8 ± 41.0 Watt to 171.2 ± 49.7 Watt in the TG compared to the SC (152.9 ± 35.6 Watt to 149.2 ± 28.4 Watt). The endurance capacity increased significantly from 17.0 ± 9.4 min to 32.8 ± 16.8 min (*p* = 0.003) in the TG compared to the SC (11.7 ± 6.2 min to 11.2 ± 7.6 min). The heart rate during the endurance test decreased in the TG from 154 ± 14 b/min to 142 ± 20 b/min for the same workload. No changes could be seen in the SC.

**Conclusion:** Endurance training in patients with moderate to severe valvular heart disease increased significantly the peakVO_2_ as well as the endurance capacity.

## Introduction

The clinical value of cardiopulmonary exercise test (CPET) offers diagnostic and prognostic information in patients with cardiovascular disease, especially concerning the peak oxygen uptake (peakVO_2_) (1–4). A higher peakVO_2_ is associated with lower cardiac mortality and morbidity in patients with cardiovascular disease (4, 5). PeakVO_2_ measured during CPET was found to objectively assess functional capacity in patients with valve disease (6–8).

Endurance training improves exercise capacity and quality of life in patients after myocardial infarction, coronary artery bypass graft surgery, and with heart failure (9–11) as well as after heart valve surgery (12–14). While exercise training is recommended in patients with moderate valve disease (15–18), the implication of training in patients with severe disease remains unclear.

The aim of this study was to investigate the effects of a 12 weeks supervised aerobic endurance training in patients with moderate to severe valve disease on the primary outcome peakVO_2_ and secondary outcomes maximum working capacity and endurance capacity.

## Methods

### Study Design and Population

A total of 37 patients with asymptomatic moderate to severe aortic valve or moderate to severe mitral valve disease attended a cardiac referral center at Medical University of Vienna over a study period of 2.5 years. Of these 6 patients declined and 1 patient was unable to exercise. Major exclusion criteria included a history of exercise- induced syncope or ventricular arrhythmias, history of hypotensive response with exercise testing (> 20 mm Hg decrease of systolic blood pressure from baseline blood pressure or an initial increase in systolic blood pressure followed by a decrease of systolic blood pressure > 20 mmHg), clinical decompensation in the previous 3 months, acute exacerbation, uncontrolled hypertension, pregnancy, inability to exercise to non-cardiovascular limitations, unwillingness to refrain from competitive sports or endurance or strength training for the duration of the study.

Thirty patients were randomly allocated to either a training group receiving 12 weeks of moderate-intensity endurance training in addition to standard care (training group, TG) or to a non-exercising control group (standard care, SC). Random allocation was performed by blocking randomization using a web-based sequence generator (http://www.randomization.com). Before randomization and after the study period of 12 weeks all patients underwent an echocardiography, a symptom limited cardiopulmonary exercise test, an endurance exercise test at 80% intensity of peakVO_2_ and a blood test. The study received approval from the local research ethics committee (EC 311/2009) and all subjects gave written informed consent.

### Echocardiographic Data

All patients underwent an echocardiographic examination by an experienced echo cardiographer. Apical 4- and 2- chamber views were used for calculation of ventricular volumes and ejections fraction using Simpson's biplane formula. Quantification of mitral regurgitation severity was based on an integrative approach including valve morphology, color Doppler jet, cavity sizes and left ventricular function. Mitral valve prolapse was defined as displacement of 1 or both leaflets by at least 2 mm below the mitral annulus level into the left atrium during systole. Flail mitral leaflet was defined as freely moving leaflet edge located in the left atrium. Systolic pulmonary artery pressure was derived from tricuspid regurgitation velocity. Aortic jet velocities were measured by continuous-wave Doppler using multiple imaging windows including the apical three and five chamber, right parasternal and suprasternal windows and taking care to record the highest velocity signal. Pressure gradients were estimated by the use of Bernoulli's equation, and aortic valve area was calculated with the continuity equation.

### Cardiopulmonary Exercise Test (CPET)

All patients underwent a symptom limited CPET on an Ergoline 800 bicycle (Sensormedics, United States) with respiratory gas-exchange analysis, using a step protocol with progressive increase in workload every minute according to a total exercise time between 8 and 12 min. The increment was adapted to the expected maximum working capacity. The CPET was performed within 3 weeks following echocardiographic examination. All patients took their medication as usual. Patients were encouraged to exercise until exhaustion. Blood pressure was measured every 2 min and continuous 12-lead electrocardiogram and oxygen saturation (SpO_2_) were recorded. Breath-by-breath minute ventilation, carbon dioxide production (VCO_2_) and oxygen uptake (VO_2_) were measured using Sensormedics 2900 Metabolic Measurement Cart. The respiratory exchange ratio (RER) was defined as VCO_2_/VO_2_, the oxygen pulse was calculated by VO_2_/heart rate and the ventilatory equivalents of VO_2_ and VCO_2_ were measured.

### Endurance Exercise Test at 80% Intensity of PeakVO_2_

An endurance exercise test was performed at an intensity of 80% of peakVO_2_. After a resting period of 3 min and a warm up period of 1 min in order to achieve the exercise workload (80% of the individual peakVO_2_), the patients were pedaling between a pedaling rate of 60 and 80/min at the given workload. The time to exhaustion, which was determined as the inability to keep a pedaling rate over 60/min, was reported. During the test every 2 min the blood pressure was measured, and continuous 12-lead electrocardiogram and oxygen saturation (SpO_2_) were recorded during the test.

### Endurance Training

The training consisted of a 3 times per week endurance training at an intensity of 60% of peakVO_2_. The training intensity was controlled by heart rate monitor (Polar Watch M420) during each training session. One training per week was performed under supervision of a physician at an outpatient clinic on an ergometer, and the other 2 training sessions per week were home-based. Oxygen saturation, blood pressure and heart rate at rest were measured before every training session and noticed by the patient and controlled every week by the physician. The training duration was between 20 and 30 min per session depending on the maximum exercise capacity tested at baseline. Every 4 weeks the training duration was increased by 15 min per week, the training intensity was unchanged during the study period of 12 weeks.

### Statistical Analysis

Calculation of sample size was based on the primary efficacy endpoint, which was defined as the change in peakVO_2_/kg between baseline and 12 weeks of exercise training. The null hypothesis to be assessed states that there is no difference in the changes of the peakVO_2_/kg value between training group and usual care group. To detect a difference in peak VO_2_/kg of 5 ml/min/kg with a standard deviation of 2 ml/kg, with a power of 80%, and a two-sided significance level of 5%, it was calculated a total sample size of 26 patients. A two-sided level of significance of α = 0.05 was used. Data were analyzed using the SPSS statistical package version 26 (IBM, New York, USA).

Normally distributed interval scaled data are presented as mean ± standard deviation (SD), ordinal scaled data as median and quartiles (25th and 75th percentile). For categorical data number of cases (n) and valid percent were (%) are shown. Between group differences between training group and standard care in baseline characteristics are estimated by *t-*test for independent samples for homogenous variances (if homogeneity is given), *t-*tests for independent samples for heterogeneous variances (if homogeneity is not given), Mann-Whitney-U-tests for ordinal data and X^2^-tests for categorial/dichotomous data. Effects of intervention (TG vs. SC) and time (baseline vs. 12 weeks) were analyzed simultaneous by General linear Models for Repeated Measurements (Analysis of Variances), while Box's-M-Test and Levene's Tests were used for proofing requirements of homogeneity of variances and covariances. Effects of time and independency between time and study group were examined via Wilk's λ. For additional examining changes within study groups, Student's *t-*test for dependent samples (for interval scaled data) and Mann-Whitney-U-test (for ordinal scaled data) were applied.

## Results

### Patients Characteristics

Patients characteristics are presented in Table 1. Thirty patients with moderate to severe asymptomatic valvular disease were included in the study, 17 patients with moderate to severe asymptomatic aortic stenosis (15 patients with severe and 2 patients with moderate-to severe aortic stenosis). Thirteen patients with moderate to severe mitral regurgitation and preserved left ventricular function (5 patients with moderate and 8 patients with severe mitral regurgitation), defined as an ejection fraction of ≥ 55 and normal sinus rhythm. The mean age of the patients was 44 ± 13 years, 17 (56.7%) were men. The baseline peakVO_2_/kg was 25.9 ± 5.3 ml/min/kg and maximum working capacity was 152 ± 37.8 Watt. The mean endurance time at baseline was 14 ± 8 min at 80% intensity of maximum workload. Medication remained unchanged during the study period. Eleven patients were on ACE (angiotensin converting enzyme) inhibitor or angiotensin II inhibitor therapy, 7 patients on betablocker therapy, 7 patients on statin, 4 patients on acetylsalicylic acid and 2 patients on metformin therapy. None of the patients had significant coronary heart disease.

**Table 1:** Baseline characteristics of study population.

	**Training group**	**Usual care**	***p-*value**
Participants, n	16	14	
Age, mean (SD), y	45 (14.4)	43.6 (12.5)	0.779
Female sex, n (%)	7 (43.8)	6 (42.9)	0.491
Weight, mean (SD), kg	73.3 (12.0)	72.4 (16.1)	0.117
Height, mean (SD), cm	173 (7.8)	173 (9.2)	0.984
Peak VO_2_m (SD), ml/min	27.2 (5.9)	24.6 (4.4)	0.214
Peak Wattm (SD), Watt	151.8 (41.0)	152.9 (35.6)	0.886
Mitral insufficiency, n (%)	7 (43.8)	6 (42.9)	0.648
Aortic stenosis, n (%)	9 (56.3)	8 (57.1)	0.648
Aortic insufficiency, n (%)	1 (6.3)	1 (7.1)	0.552
Hypertension, n (%)	3 (18.8)	7 (50.0)	0.450
Diabetes mellitus, n (%)	1 (6.3)	1 (7)	0.704
Dyslipidaemia, n (%)	5 (31)	4 (29)	0.567
Active Smoking, n (%)	1 (6)	1 (7.1)	0.724
LVEF% (SD)	60.3 (4.3)	61.2 (5.7)	0.631
Vmax, m/s (SD)	3.5 (1.5)	3.3 (1.7)	0.807
NTproBNP, pg/l	99.0 (37–339)	96.4 (71–248)	0.837

*VO_2_m, mean oxygen uptake; Wattm, mean workload; LVEF, left ventricular ejection fraction; Vmax, maximum transvalvular flow velocity; NTproBNP, N-terminal probrain natriuretic peptide. Categorical data are expressed as n (%), interval scaled data as mean (SD, standard deviation)*.

After baseline assessment 16 patients were randomized to the training group, 9 patients with aortic stenosis, and 7 patients with mitral regurgitation. One of the patients with aortic stenosis had severe aortic stenosis and aortic regurgitation. Fourteen patients were randomized to the standard care group, eight patients with severe aortic stenosis, 6 patients with mitral regurgitation. One of the patients with aortic stenosis had moderate aortic stenosis and aortic regurgitation. There were no significant differences for the baseline characteristics between both groups.

### Outcomes

#### Primary Endpoint: Peak Oxygen Uptake (PeakVO_2_)

Primary endpoint was the change in peakVO_2_ ml/min/kg. A significant effect of intervention and time (baseline vs. 12 weeks) for peakVO_2_ ml/min/kg could be shown (Table 2). The peakVO_2_ ml/min/kg was significantly higher in the TG after the study period of 12 weeks (peakVO_2_ ml/min/kg 30.4 **±** 6.3 ml/min/kg) compared to the SC (peakVO_2_ ml/min/kg 24.7 **±** 3.8 ml/min/kg *p* < 0.007). PeakVO_2_ ml/min/kg increased in the TG from 27.2 **±** 5.9 to 30.4 **±** 6.3 ml/min/kg, mean increase relative to baseline +16.5%, *p* < 0.001, no significant changes between the peakVO_2_ ml/min/kg could be seen in the SC (peakVO_2_ ml/min/kg from 24.6 **±** 4.4 to 24.7 **±** 3.8 ml/min/kg, *p* = 0.916) after the 12-weeks study period (Table 2).

**Table 2:** Parameters at baseline and after training period.

	**Training group (TG)**			**Standard care (SC)**			ANOVA time*group
	**Baseline**	**End 12-week training**	*p*-value*	**Baseline**	**End 12-week training**	*p*-value**	***p*-value**
peakVO_2_ ml/min/kg (SD)	27.2 (5.9)	30.4 (6.3)	**0.001**	24.6 ± 4.4	24.7 ± 3.8	0.768	**0.007**
Wattmax Watt (SD)	151.8 (41.0)	171.2 (49.7)	**0.017**	152.9 ± 35.6	149.2 ± 28.4	0.321	**0.001**
Wattmax Watt/kg (SD)	2.08 (0.48)	2.45 (0.53)	**0.001**	1.9 ± 0.54	1.9 ± 0.52	0.456	**0.001**
VE/VCO_2_ slope (SD)	21.8 (3.7)	22.4 (2.6)	0.854	26.4 ± 1.5	25.6 ± 1.6	0.642	0.768
Lactate mmol/L (SD)	7.7 (2.3)	7.4 (2.4)	0.954	6.8 ± 2.0	7.3 ± 2.3 *	0.670	0.630
RER (SD)	1.2 (0.2)	1.2 (0.1)	0.150	1.1 ± 0.1	1.1 ± 0.1	0.680	0.172
Oxygen pulse ml/min (SD)	12.2 (2.6)	13.8 (3.1)	0.017	12.5 ± 3.9	13.4 ± 3.6	**0.033**	0.180
HR max beats/min (SD)	161 (19)	158 (19)	0.181	163 ± 14	159 ± 19	0.257	0.147
Endurance time min (SD)	17.0 (9.4)	32.8 (16.8)	**0.001**	11.7 ± 6.2	11.2 ± 7.6	0.850	**0.003**
HR submaximal test (SD)	154 (14)	142 (20)	**0.014**	159 ± 20	159 ± 24	0.443	0.147
NT-proBNP pg/l	99.0 (37–339)	200 (96–506)	0.905	96.4 (71–248)	113 (102–221)	0.064	0.642
LVEF% (SD)	60.3 (4.3)	60.5 (3.0)	0.821	61.2 (5.7)	60.1 (3.5)	0.570	0.558

*peakVO_2_, peak oxygen uptake; Wattmax, maximum working capacity; VE/VCO_2_slope, ventilatory equivalent for carbon dioxide-slope; RER, respiratory exchange ratio; HR max, maximum heart rate; HR, heart rate; NT-proBNP, N-terminal probrain natriuretic peptide; Data are expressed as mean (SD) with the exception of NT-proBNP (median, 25th−75th percentile)*.

p-value^*^, p-value^**^-changes within study groups applied by Student's t-test for dependent samples (for interval scaled data) and Wicoxon-test, ANOVA- effects of intervention (TG vs. SC) and time (baseline vs. 12 weeks) were analyzed by Analysis of Variances. Bold values provide statistically significance.

#### Secondary Outcomes: Maximum Working Capacity, Endurance Capacity

Significant effects of intervention and time could be shown for maximum working capacity, Watt_max_/kg and endurance time (Table 2). Watt_max_ significantly improved in the training group after the 12 weeks (Watt_max_ = 151.8 **±** 41.0 Watt to 171.2 **±** 49.7 Watt, *p* < 0.001) compared to the control group (Watt_max_ = 152.9 **±** 35.6 Watt to 149.2 **±** 28.4 Watt, *p* < 0.410). In the training group the endurance capacity increased significantly from 17.0 **±** 9.4 s to 32.8 **±** 16.8 s (*p* < 0.004) after the 12 weeks study period. No significant changes could be observed in the control group (10.8 **±** 6 s to 11.2 **±** 7.6 s, *p* = 0.850). The submaximal heart rate at 80% intensity of maximum capacity decreased after the 12 weeks in the training group from 154.3366 **±** 14 to 141.8 **±** **20** (*p* = 0.014), no changes could be seen in the control group (158.8 ± 20 to 159.3 ±24, *p* = 0.442, Table 2). No significant changes could be seen concerning the NT-proBNP levels between both groups.

## Discussion

In this randomized controlled trial, we investigated the effect of a 12 week endurance training compared to standard care in patients with moderate to severe heart disease. Thereby we could show that the exercise training can increase the peakVO_2_ and the endurance capacity significantly in these patients without adverse events over a training period of 12 weeks. Moreover, no significant worsening in echocardiography could be observed after the training period with no adverse events during the training sessions or significant changes of NT-proBNP levels. Our findings are in accordance with previous studies demonstrating that physical exercise training can improve peakVO_2_ in patients with valvular heart disease after heart valve surgery (11–14). Whereas, there are strong recommendations concerning exercise training in mild to moderate valvular disease, there are no clear recommendations regarding exercise training in asymptomatic moderate to severe valvular disease (15–19). In previous studies a relationship between peakVO2 and the prognosis and mortality of patients with valvular heart disease has been reported (4–6). Defining the optimal timing of surgery in severe valvular heart disease is often difficult, but abnormal exercise response, such as symptoms during exercise, is one of the parameters which is taken into account for the surgery time point (7, 8). Improvement of peakVO2 may therefore be strongly related to the prognosis of these patients and the course of the disease. While peakVO_2_ describes the net limitation in exercise capacity, submaximal tests such as endurance tests at 80% of maximum capacity often correlated more with activities of daily livings.

To our knowledge, no previous trials have investigated the effects of an endurance training in patients with moderate to severe valvular heart disease concerning the peak VO_2_ and submaximal endurance performance in patients with moderate to severe valvular disease.

Several limitations have to be noted, the most important was the low sample size. However, the trial was designed to be more explorative rather than confirmative. The study may result in further investigational studies with larger sample sizes. Further there might be selection bias as only highly selected individuals accept participation, which is common in training studies. Another possible limitation is the missing evaluation of quality of life, which could support the efficacy of endurance training, even though all the patients were asymptomatic at baseline, during the stress test and the study period.

In summary, in this study it could be shown, that a home-based exercise training combined with a part-time supervision is safe and effective in these patients and can improve the peak VO_2_ and the endurance capacity significantly in patients with moderate to severe valvular disease.

## Data Availability Statement

The original contributions presented in the study are included in the article/supplementary material, further inquiries can be directed to the corresponding author/s.

## Ethics Statement

The studies involving human participants were reviewed and approved by Ethics committe Medical University of Vienna. The patients/participants provided their written informed consent to participate in this study.

## Author's Note

Peak oxygen uptake (peakVO2) is one of the strongest predictors of survival in patients with valvular heart disease. In patients with mild to moderate disease endurance training is strongly recommended for prognosis and the course of the disease. Whereas, symptom limited cardiopulmonary exercise test is usually performed in patients with severe valvular heart disease there are still concerns how to exercise these patients. Symptoms like dyspnea on exertion are parameters taken into account for the surgery time point, but they may be non-specific and due to inactivity. Endurance training and improvement of peakVO2 in patients with severe valvular heart disease could not only improve the exercise capacity of these patients but can also provide more information about the functional status and prognosis.

## Author Contributions

KV designed the study, performed study examination, acquired data, analyzed data, and wrote the manuscript draft. DH performed study examination, acquired, and analyzed the data. RR, MS, SA, and HG performed study examination and acquired data. RR and HG designed the study. All listed authors read, revised, and finally approved the manuscript.

## Conflict of Interest

The authors declare that the research was conducted in the absence of any commercial or financial relationships that could be construed as a potential conflict of interest.
